# A potentiated cooperation of carbonic anhydrase IX and histone deacetylase inhibitors against cancer

**DOI:** 10.1080/14756366.2019.1706090

**Published:** 2019-12-22

**Authors:** Jessica Ruzzolini, Anna Laurenzana, Elena Andreucci, Silvia Peppicelli, Francesca Bianchini, Fabrizio Carta, Claudiu T. Supuran, Maria Novella Romanelli, Chiara Nediani, Lido Calorini

**Affiliations:** a Department of Experimental and Clinical Biomedical Sciences “Mario Serio”, University of Florence, Florence, Italy; b Department of NEUROFARBA, University of Florence, Florence, Italy; c Center of Excellence for Research, Transfer and High Education, DenoTHE University of Florence, Florence, Italy

**Keywords:** Carbonic anhydrase IX, SLC-0111;SAHA, histone acetylation, combined therapy

## Abstract

The emergence of tumour recurrence and resistance limits the survival rate for most tumour-bearing patients. Only, combination therapies targeting pathways involved in the induction and in the maintenance of cancer growth and progression might potentially result in an enhanced therapeutic efficacy. Herein, we provided a prospective combination treatment that includes suberoylanilide hydroxamic acid (SAHA), a well-known inhibitor of histone deacetylases (HDACs), and SLC-0111, a novel inhibitor of carbonic anhydrase (CA) IX. We proved that HDAC inhibition with SAHA in combination with SLC-0111 affects cell viability and colony forming capability to greater extent than either treatment alone of breast, colorectal and melanoma cancer cells. At the molecular level, this therapeutic regimen resulted in a synergistically increase of histone H4 and p53 acetylation in all tested cell lines. Overall, our findings showed that SAHA and SLC-0111 can be regarded as very attractive combination providing a potential therapeutic strategy against different cancer models.

## Introduction

Despite the sharp increase of therapeutic options, cancer remains the second leading cause of death worldwide^1^^,^^2^. Indeed, although selective drugs targeting oncogenic driver mutations are initially highly effective, acquired resistance to chemo- and target-therapy occurs and, often results in tumour relapse and low quality of patient life^3–6^. Nonetheless, the rationale for most cancer therapeutic strategies is to target malignant cancer cells while largely ignoring tumour microenvironment in which they are endowed^7^^,^^8^. The fast proliferation of tumour cells, due to the accumulation of genetic and epigenetic alterations, rapidly results in a limitation of oxygen availability and ultimately hypoxia^9^^,^^10^. Hence, hypoxia triggers several events such as metabolic reprogramming of tumour cells, and modulation of tumour microenvironment (TME) that results in the rearrangement of extracellular matrix and formation of new faulty and leaky vessels, which may lead to metastasis^11^. In order to survive in an oxygen deficient environment, tumour cells reprogramme their metabolism to an anaerobic glycolysis resulting in the intracellular accumulation of lactic and carbonic acid^12–16^. To avoid the toxic intracellular acidification, tumour cells potentiate the expression of extrusion mechanisms, including monocarboxylate transporters and proton flux regulators, such as vacuolar H^+^-ATPases, Na^+^/H^+^ exchanger, Na^+^/HCO_3_ co-transporter and carbonic anhydrase (CA) IX^17–20^. In particular, CA IX, catalysing the reversible conversion of carbon dioxide to a proton and bicarbonate and thereby neutralising the acidic conditions, allows tumours (i) to survive in a hostile environment with low oxygenation and low pH, and hence (ii) to resist to chemo and radiotherapy and (iii) to suppress anticancer immune responses. Indeed, increased expression of CA IX has been shown in a wide spectrum of tumour histo-types compared with normal tissues. Since several clinical studies show a clear relationship between high CA IX levels in tumours and a poor prognosis, CA IX is likely an attractive target for cancer therapy^21–25^. Indeed, CA inhibitors have been shown to elicit synergistic effects when used in combination with other chemotherapeutic agents, thus represents a well-suited agent to use alongside conventional therapy: this is the so-called complementary therapy of cancer. Our earlier studies showed that SLC-0111, a novel CA IX inhibitor, is able to synergize with cytotoxic drugs such as Dacarbazine, Doxorubicine and 5-Fluorouracil in melanoma, breast and colorectal cancer cell lines, which express high levels of CA IX also in normoxia^26^.

Given the fact that the one of the most common epigenetic alteration in tumour onset and progression is the overexpression or the aberrant recruitment of histone deacetylases (HDACs) to the promoter of tumour suppressor genes^27–29^, HDACs have been considered as therapeutic targets for the treatment of cancer. HDACs deacetylate the lysine residues on histones leading to the inaccessibility of transcriptionally active regions of DNA. HDACs are also responsible for removing the acetyl group from non-histone proteins such as p53 and thus, interfering with several cellular process such as DNA damage repair, protein stability and DNA binding activity of transcriptional factors. According to the Human Protein Atlas HDACs are expressed in a variety of tumors^30–35^ and some HDAC isoforms such as HDAC2 was found expressed in 100% of multiple tumors^36^. As a consequence, histone deacetylase inhibitors (HDACi) have emerged as important agents for cancer treatment due to their multiple anti-cancer effects, such as the ability to induce differentiation, cell cycle arrest, apoptosis and inhibit angiogenesis^37–41^. However many HDACi are very efficient *in vitro*, when given as monotherapy, but may be toxic *in vivo* at therapeutic levels and their use is recommended in patients who had failed or relapsed from standard therapy. To date, suberoylanilide hydroxamic acid (SAHA), a second generation HDAC inhibitor, has shown to arrest cell cycle progression and promote cancer cell apoptosis *in vitro* on different solid tumours while its use in clinical trials is limited for the treatment of recurrent T-cell lymphoma^42^.

Currently, there is a great interest in developing combined approaches aiming to create synergistic or additive effects and thus, to improve the therapeutic index avoiding adaptative resistance and toxic effects. Herein, we report the antiproliferative effects of SAHA in combination with SLC-0111 on breast, colorectal and melanoma cancer cells. We proved that HDAC inhibition in combination with SLC-0111 affects either short-term and long-term cell proliferation to greater extent than either treatment alone causing a synergistic increase of H4 and p53 acetylation in all tested cell lines. Our findings provided a new potential therapeutic strategy of SAHA and CA IX inhibition in different cancer models.

## Materials and methods

### Cell lines and culture conditions

In this study, we used A375M6, isolated in our laboratory from lung metastasis of SCID bg/bg mice i.v. injected with A375 human melanoma cell lines, obtained from American Type Culture Collection (ATCC, Rockville, MD), human colorectal carcinoma cell line HCT116, a kind gift of Dr. Matteo Lulli, Department of Clinical and Experimental Biomedical Sciences, University of Florence and human breast carcinoma MCF7 (from ATCC). Cells were supplemented with 10% foetal bovine serum (FBS, Euroclone, MI, Italy), at 37 °C in humidified atmosphere containing 90% air and 10% CO_2_. Viability of the cells was determined by trypan blue exclusion test. Cultures were periodically monitored for mycoplasma contamination using Chen’s fluorochrome test. According to the experiments, cells were treated with a CA IX inhibitor, SLC-0111, developed in the laboratory of Prof. C.T. Supuran^22^ alone or in combination with SAHA (from Sigma-Aldrich, Milan, Italy).

### MTT assay

Cell viability was assessed using MTT (3–(4,5-dimethylthiazol-2-yl)-2,5-diphenyltetrazolium bromide) tetrazolium reduction assay (Sigma Aldrich, Milano). Cells were plated into 96-multiwell plates in complete medium without red phenol. FC16 and SAHA were added to the medium colture for 72 h. Then the MTT reagent was added to the medium and plates were incubated at 37 °C. After 2 h, MTT was removed and the blue MTT–formazan product was solubilised with Dimethyl sulfoxide (DMSO) (Sigma Aldrich, Milano). The absorbance of the formazan solution was read at 595 nm using the microplate reader (Bio-Rad).

### Cell cycle analysis

Cell cycle distribution was analysed via the DNA content using the PI staining method. Cells were centrifugated and stained with a mixture of 50 µg/mL PI (Sigma-Aldrich, St. Louis, MO, USA), 0.1% trisodium citrate and 0.1% NP40 (or triton x-100) (Sigma-Aldrich, St. Louis, MO, USA) in the dark at 4 °C for 30 min. The stained cells were analysed via flow cytometry (BD-FACS Canto, BD Biosciences, Franklin Lakes, NJ, USA) using red propidium-DNA fluorescence.

### Plate colony forming assay

Approximately 100 cells/mL were seeded in fresh medium, and incubated at 37 °C. The following day cells were treated with drugs and incubated at 37 °C for two weeks, during which treatment was repeated two times. After two weeks cells were washed with PBS, fixed in cold methanol, and stained using a Diff Quik kit (BD Biosciences). The stained colonies were photographed with a digital camera and the number of colonies in each well was counted.

### Western blotting analysis

Cells were washed with ice cold PBS containing 1 mM Na_4_VO_3_, and lysed in cell RIPA lysis buffer (Merk Millipore, Vimodrone, MI, Italy) containing sodium orthovanadate (Sigma-Aldrich) and protease inhibitor (Life Technologies, Monza, Italy). Aliquots of supernatants containing equal amounts of protein (30 µg) in Laemmli buffer were separated on Bolt^®^ Bis-Tris Plus gels 4–12% precast polyacrylamide gels (Life Technologies, Monza, Italy). Fractionated proteins were transferred from the gel to a PVDF nitrocellulose membrane using iBlot 2 system (Life Technologies, Monza, Italy). Blots were stained with Ponceau red to ensure equal loading and complete transfer of proteins, then they were blocked for 1 h, at room temperature, with 5% milk in PBS 0.1% tween solution. Subsequently, the membrane was probed at 4 °C overnight with the following primary antibodies; rabbit anti-PARP (Cell Signaling); rabbit anti-acetylated Histon4 (Upstate), rabbit anti acetylated p53 (Upstate); while rabbit anti-GAPDH (cell Signaling) was used to assess equal amount of protein loaded in each lane. Anti-Rabbit IgG (whole molecule)–Peroxidase antibody (Sigma) has been used as secondary antibodies; the ECL procedure was employed for development.

## Results

### Dual HDAC and CA IX targeting reduces the growth of A375M6, HCT116 and MCF7 cancer cells

**Figure 1.**
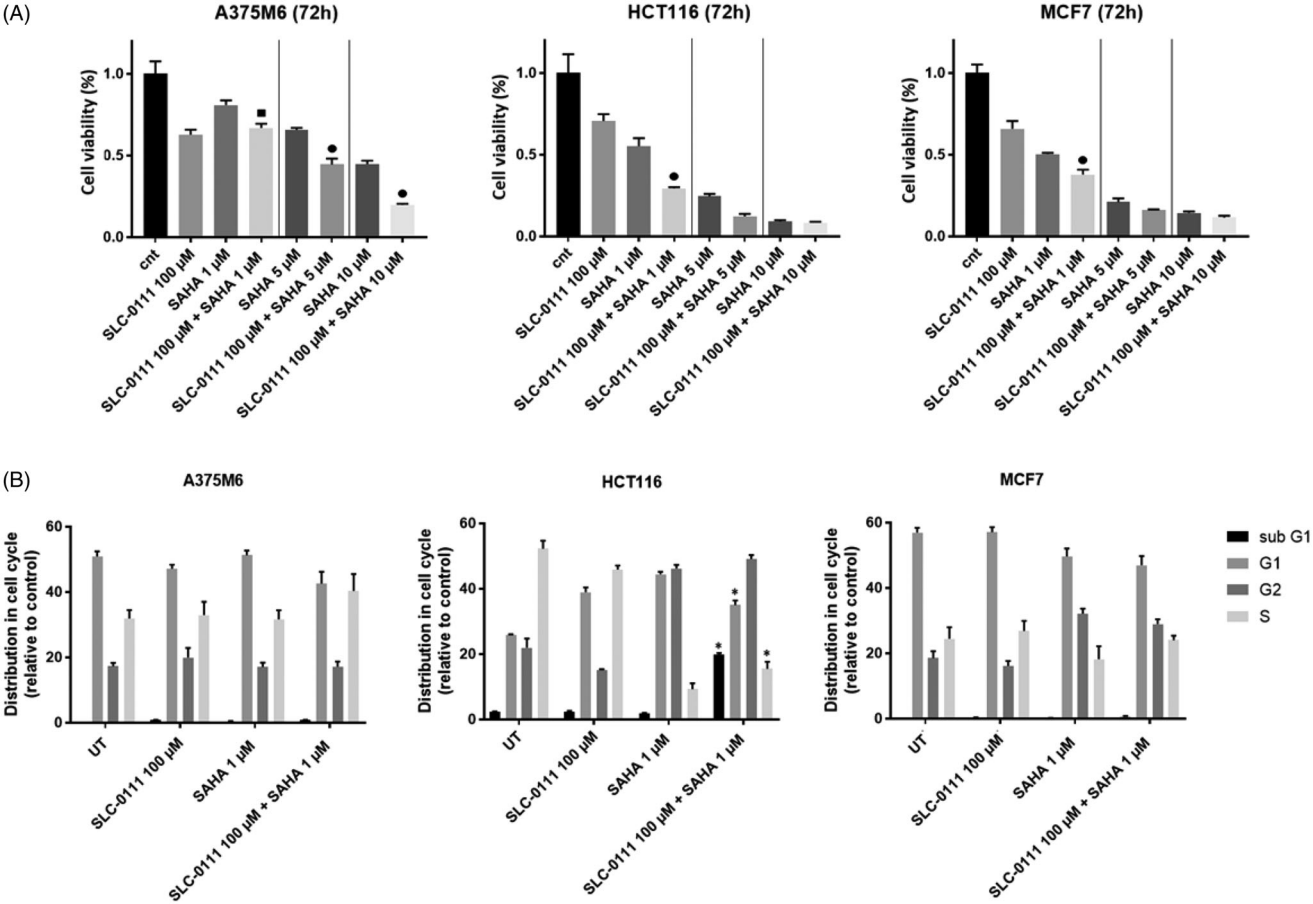
SAHA- SLC-0111 efficacy on A375M6, HCT116 and MCF7. (A) Cell viability after 72 h treatment of SLC-0111 alone or in combination with three doses of SAHA evaluated by MTT assay; ■ *p* ≤ .05 refers to the co-treatment SLC-0111 + SAHA respect to SAHA alone; ● *p* ≤ .05 refers to the co-treatment SLC-0111 + SAHA respect to SAHA and SLC-0111 alone. (B) Cell cycle distribution analysed by FACS; *p* ≤ .05 refers to the co-treatment SLC-0111 + SAHA respect to SLC-0111 alone.

We explored the anti-tumour activity of the histone deacetylase (HDAC) inhibitor, suberoylanilide hydroxamic acid (SAHA) alone or in combination with the CA IX inhibitor, SLC-0111, across panel of cancer cell lines. The MTT cell proliferation assay was performed to monitor the cell viability of A375-M6 melanoma, HCT116 colorectal, MCF7 breast cancer cells treated for 72 h with increasing doses of SAHA alone or in combination with a fixed dose of SLC-0111, which was determined in a previous study^26^. As shown in Figure 1(A) SAHA inhibited cell proliferation in a dose-dependent manner in all cell lines whereas combination of SAHA and SLC-0111 significantly enhanced the effectiveness of SAHA at the lowest dose. Of note, HCT116 and MCF7 shared similar relative sensitivity to these two inhibitors, while A375-M6 cells were less responsive to SAHA. Similar differences across cell lines were also observed using other cell proliferation assay such as cell cycle distribution. To characterise whether SAHA caused cells to arrest in a specific cell cycle phase, A375M6, HCTC116 and MCF7 cells were treated with the lowest tested dose of SAHA for 72 h. Cell cycle distribution analysis in Figure 1(B) showed that SAHA alone and in combination with SLC-0111 increased the percentage of cells in G2/M phase to about 20% in HCT116 and 10% in MCF7 cells (Figure 1(B)), while no significant changes in cell cycle distribution were observed for A375M6 cells. Moreover, FACS analysis revealed that the combo treatment SAHA plus SLC-0111 was able to induce an increase of the percentage of HCT116 cells in the sub-G1 phase indicative of cell death, thus confirming the highest sensitivity of this cell line to the drug combination.

### SAHA/SLC-0111 combined treatment completely restrained the colony formation

**Figure 2.**
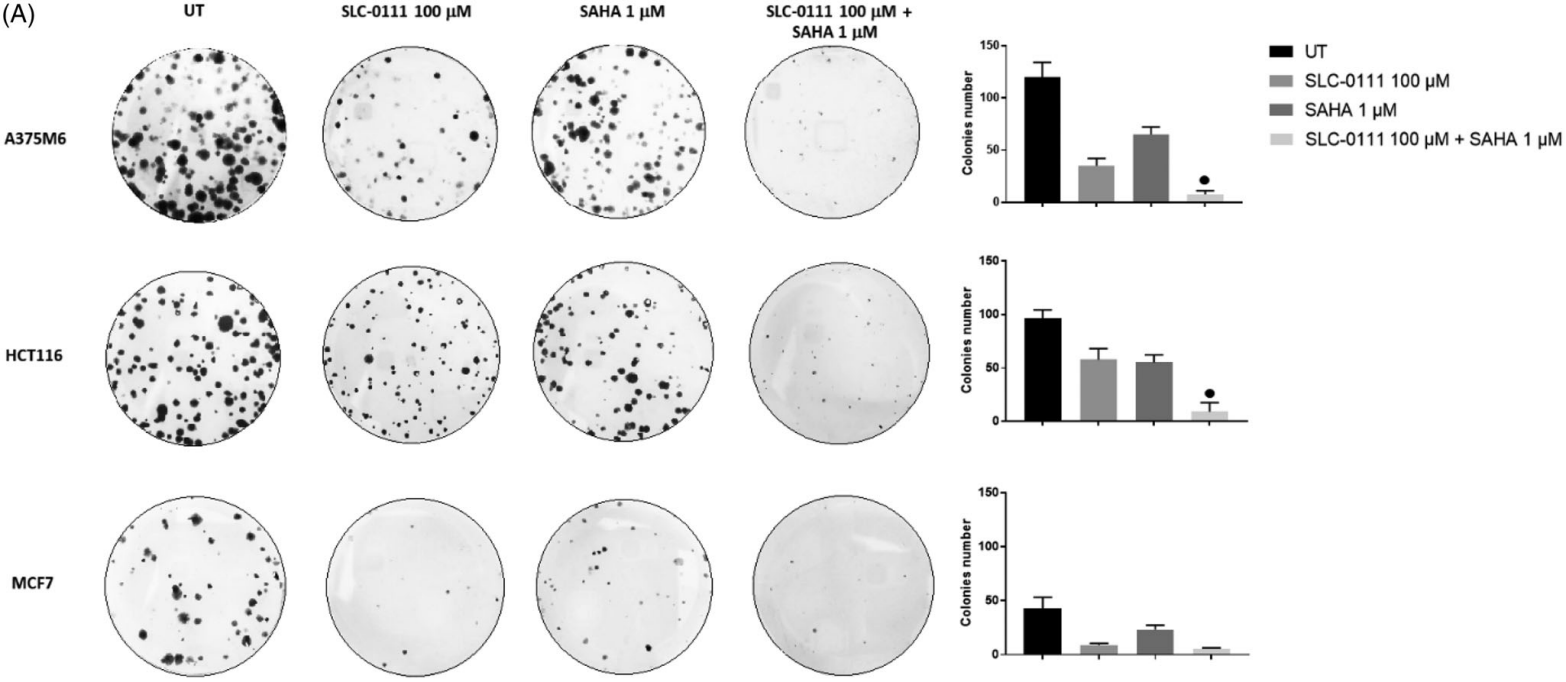
SAHA- SLC-0111 efficacy on the ability of A375M6, HCT116 and MCF7 to form colonies. (A) Colony Forming Units (CFU) assay of cells treated with SLC-0111 and/or SAHA; ● *p* ≤ .05 refers to the co-treatment SLC-0111 + SAHA respect to SAHA and SLC-0111 alone.

We next performed a clonogenicity assay on A375M6, HCT116 and MCF7 cells treated either with SLC-0111, SAHA or the combination. Colonies were defined as cluster of 20 cells or more after 14 days. While colony formation was significantly reduced by SLC-0111 or SAHA alone, combination treatment almost completely abrogated the capability to form colonies (Figure 2(A)) of all three cancer cell lines. It is worth noting the synergistic effect of the two inhibitors on HCT116 cells and the strong sensitivity of MCF7 to the single agents alone.

### Enhanced acetylation of p53 and H4 after dual HDAC and CA IX inhibition

**Figure 3.**
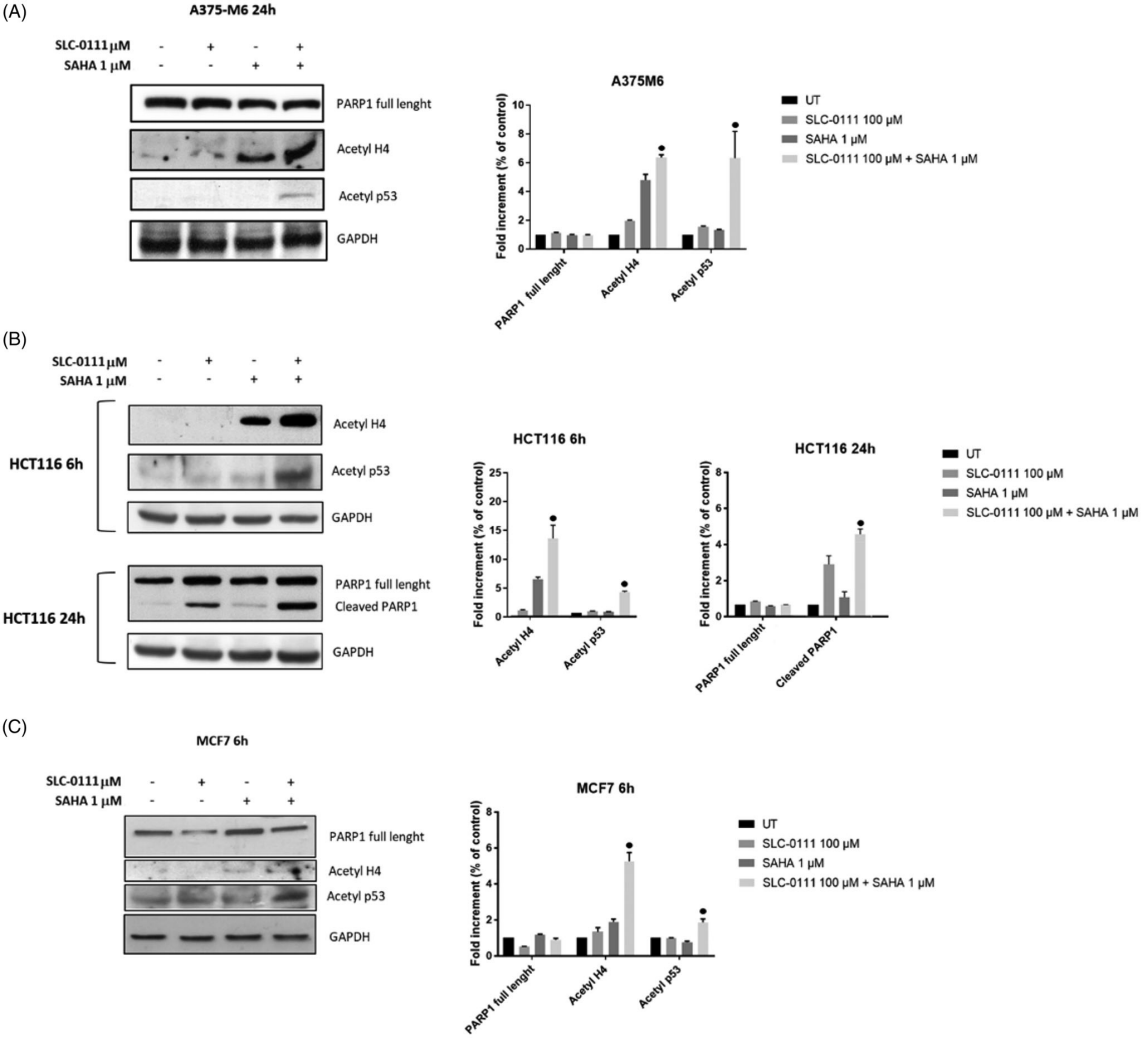
Molecular effect after the treatment with SLC-0111 and/or SAHA. (A) (Left) Representative Western blot of PARP1, Acetyl H4, Acetyl p53 after the treatment of A375M6 with SLC-0111 and/or SAHA. (Right) Densitometric quantification of PARP1, Acetyl H4, Acetyl p53 relative to GAPDH expression, expressed as a fold increment (%) compared to UT. ● *p* ≤ .05 refers to the co-treatment SLC-0111 + SAHA respect to SAHA and SLC-0111 alone. (B) (Left) Representative Western blot of Acetyl H4 and Acetyl p53 after the treatment of HCT116 with SLC-0111 and/or SAHA for 24 h and representative Western blot of PARP1 and cleaved PARP1 after the treatment of HCT116 with SLC-0111 and/or SAHA for 24 h. (Right) Densitometric quantification of Acetyl H4, Acetyl p53, PARP1 and cleaved PARP1 relative to GAPDH expression, expressed as a fold increment (%) compared to UT. ● *p* ≤ .05 refers to the co-treatment SLC-0111 + SAHA respect to SAHA and SLC-0111 alone. (C) (Left) Representative Western blot of PARP, Acetyl H4 and Acetyl p53 after the treatment of MCF7 with SLC-0111 and/or SAHA for 6h. (Right) Densitometric quantification of PARP1, Acetyl H4 and Acetyl p53 relative to GAPDH expression, expressed as a fold increment (%) compared to UT. ● *p* ≤ .05 refers to the co-treatment SLC-0111 + SAHA respect to SAHA and SLC-0111 alone.

In order to illustrate the molecular basis of the inhibitor interaction, we performed western blot analysis for acetylated histone H4 and acetylated not histone protein such as p53, which are target proteins of SAHA activity. We remarkably found a synergistic increase of H4 and p53 acetylation on all three cancer cell lines after the combo treatment (Figure 3(A)). To further investigate the mechanism involved in apoptosis induction on HCT116 cells, the cleavage of poly (ADP-ribose) polymerase (PARP) was studied upon treatment with either single inhibitors alone or in combination. PARP cleavage by activated caspase-3 is known as an early marker for apoptosis induction. Western blot images and analyses showed that cleaved PARP was observed after treating HCT116 cells with either SAHA or SLC-0111 alone, while was enhanced in presence of both inhibitors. These data were consistent with the increase of sub G1 population induced by the combo treatment and measured by FACS analysis. No detectable cleaved fragments were observed on A375M6 and MCF7 cells.

## Discussion and conclusions

The combination of two or more drugs targeting different cancer pathways is now being actively pursued^43–46^. This approach creates synergistic effects able to reduce the emergence of tumour recurrence and resistance, since cancer cells are most likely incapable of adapting to the simultaneous effects of two therapeutic agents^47–51^. However, a combination therapy does not exclude side effects in treated patients, disclosing the need for a combination therapy made by standard chemotherapy and biological drugs. In this study, we provide a prospective combination of treatment that includes an inhibitor of histone deacetylases (HDACs), as their dysregulation is associated with many forms of cancers^29–36^, and an inhibitor of CA IX, as its overexpression in various tumours is correlated with cancer progression and poor survival^17^^,^^52^. We have previously reported that melanoma, breast and colorectal cancer cell lines, express high levels of CA IX in normoxia whereas the transiently and chronically exposure to extracellular acidic microenvironment (pH 6.7 ± 0.1) induces CA IX overexpression^53^. Moreover, we demonstrated that SLC-0111, a novel CA IX inhibitor, is able to synergize with cytotoxic drugs such as Dacarbazine, Doxorubicine and 5-Fluorouracil^26^.

In the past 20 years, histone deacetylase inhibitors (HDACi), have emerged as promising chemotherapeutic agents in pre-clinical and clinical trials. The specific appeal of HDACi for tumour treatment is due to their ability to induce differentiation, cell cycle arrest, apoptosis, autophagy and restrain angiogenesis. The specific function of HDACi is to inhibit the activity of histone deacetylases, enzymes that deacetylate the lysine residues on histone molecules and as well as on non-histone proteins, thus removing the transcriptional repression^28^^,^^54^. Trichostatin A was the first HDACi tested in clinical trials while SAHA, a potent inhibitor of HDAC1, HDAC2, HDAC3 and HDAC6, was the first FDA-approved HDACi for the treatment of recurrent cutaneous T-cell lymphoma^42^. In preclinical setting, SAHA has been shown to inhibit tumour growth, to induce cell cycle arrest, differentiation or apoptosis in a variety of transformed cell lines, including breast cancer cells^54^^,^^55^. Depending on dose, time of treatment, and cell line, SAHA can induce a G1 or G2/M cell cycle arrest. Furthermore, a recent study unveiled that the tumour selective action of SAHA is associated with compromised DNA repair mechanism, such as a defective checkpoint kinase 1 (Chk1) in several cancer cells^56^^,^^57^. Thus, the preferential efficacy of HDACi on cancer cells and their capability to synergistically enhance the anti-tumour potential of chemotherapeutic agents have prompted numerous preclinical and clinical investigations.

In the current study, we found that the administration of CA IX inhibitor potentiated the antitumor activity of SAHA in several cancer lines. Notably, we discovered that the combination of SLC-0111 (100 µM) and SAHA (1 µM) displayed significantly higher effects on cell viability and colony forming capability, compared to either agent alone for the treatment of melanoma, breast and colorectal cancer. Moreover, according to FACS analysis we observed, that cell cycle changes induced by single or combined HDACi/CA IXi treatment depended on the cell line used. In particular, we were able to detect a distinctly enhanced apoptosis only on colon cancer cells after the 24 h combined treatment. The apoptotic effects were confirmed by the induction of PARP cleavage.

At the molecular level we proved that the synergistic interactions between the two inhibitors was associated to the enhanced acetylation of H4 and p53, that is one of the first recognised and important non-histone proteins targeted by HDACs. It has been demonstrated that p53 regulates cell apoptosis and autophagy among its multitude of other functions as the “gatekeeper” of the cell. Since p53 acetylation is crucial for its transcription-independent pro-apoptotic functions, the higher p53 acetylation level in HCT116 compared to the other cell lines might also explain why single and combined treatments are effective in inducing apoptosis.

Overall, our findings along with the results obtained in other studies^58^ showed that the CA inhibitor with HDACi can be regarded as a very attractive combination for the treatment of different types of tumours. In the current study, the combined treatment beyond exerting anti-proliferative effects on several cancer cell lines, has also the ability of inducing *in vitro* apoptosis of colon cancer cells. Moreover, a highly effective synergism between the two molecules might help in decreasing SAHA concentrations at levels that might be less toxic, thus offering a new promise in the combined pharmacological treatment of various tumour histo-types, escaping side effects in the patients.

Although some hydroxamates have been reported to possess significant CA inhibitory properties,^59–61^ SAHA is not among these derivatives. Indeed, against isoforms CA IX and XII SAHA showed no inhibition up to 50 µM (unpublished results from the authors’ laboratories). Thus, the synergy reported in the present work is indeed due to the concomitant inhibition of the tumour associated CA isoforms (e.g. CA IX) by the sulphonamide derivative SLC-0111, and of the HDAC isoforms involved in tumorigenesis by SAHA.
